# Efficacy of Sutureless Scleral Fixation of One-Piece T-Shaped Haptic Intraocular Lens in Maintaining Anterior Chamber Stability During Descemet Membrane Endothelial Keratoplasty in Vitrectomized Eyes: Leak Test and Iris Diaphragm Reconstruction

**DOI:** 10.3390/jcm13226654

**Published:** 2024-11-06

**Authors:** Agostino Salvatore Vaiano, Antonio Greco, Maria Marenco, Andrea Greco, Alessandro De Filippis, Fabio Garavelli, Riccardo Merli, Vito Romano

**Affiliations:** 1 Institute of Ophthalmology, Santa Croce e Carle Hospital, Via Michele Coppino 26, 12100 Cuneo, Italy; antonio.greco89@gmail.com (A.G.); marencomaria9012@gmail.com (M.M.); a.greco88.ag@gmail.com (A.G.); defilippis.ale@gmail.com (A.D.F.); fabio.garavelli@libero.it (F.G.); riccardomerli01@gmail.com (R.M.); 2 Eye Unit, Department of Medical and Surgical Specialties, Radiological Sciences, and Public Health, University of Brescia, Viale Europa 15, 25123 Brescia, Italy; vito.romano@unibs.it

**Keywords:** scleral lens fixation, DMEK surgery, complication, graft dislocation, graft detachment, IOL damage, IOL opacification

## Abstract

**Objectives**: This study aimed to describe the outcomes of a staged procedure combining Descemet membrane endothelial keratoplasty (DMEK) and sutureless scleral fixation (SSF) of a one-piece intraocular lens (IOL) in a case series. Co-performing endothelial keratoplasty (EK) and SSF is associated with intraoperative and postoperative complications such as graft deployment difficulties, air migration, graft detachment, and IOL opacification or tilt, all of which are evaluated in this study. **Methods:** This is a retrospective observational case series. Clinical data were collected from eight eyes of eight patients who underwent DMEK for endothelial failure and had previously received an SSF with one-piece IOL following complete vitrectomy. During DMEK surgery, an air leak test was conducted to check for air migration into the posterior chamber. If instability was detected, pupilloplasty was performed. Intraoperative and postoperative data, including DMEK graft unfolding time, were collected. Corrected Distance Visual Acuity (CDVA), refraction, endothelial cell density (ECD), central corneal thickness (CCT), intraocular pressure (IOP), and complications were recorded over a 12-month follow-up period. **Results:** We performed pupilloplasty in four patients (50%). The median CDVA improved from preoperative 0.85 logMAR (range: 0.60 to 1.00) at baseline to 0.18 logMAR (range: 0.10 to 0.70, *p* = 0.012) at 12 months. The median refraction value changed significantly from −1.00 to −0.50 at 12 months. The median percentage reduction in ECD after 12 months was 33.4% (range 30 to 40). The median baseline CCT was 689 μm (range: 651 to 701) at baseline visit and 541.5 μm (range: 525 to 591, *p* = 0.008) at 12 months. The median IOP was reduced significantly during follow-up. The median graft unfolding time was 6 min (5 to 9). One patient required rebubbling for partial detachment on postoperative day one. No complications occurred within 12 months. **Conclusions:** The effective compartmentalization of the anterior and posterior chambers in vitrectomized eyes with an SSF one-piece IOL and pupilloplasty can facilitate critical steps of DMEK surgery in complex eyes. Additionally, the air leak test could prove useful in identifying the need for iris-lens diaphragm reconstruction.

## 1. Introduction

Descemet membrane endothelial keratoplasty (DMEK) has emerged as the gold-standard surgical intervention for addressing endothelial failure [1]. Despite the predictability and reproducibility of DMEK, its application can be particularly challenging in complex cases, such as those involving vitrectomized eyes with scleral lens fixation [2,3]. The primary challenge in vitrectomized eyes stems from the necessity of creating a shallow and stable anterior chamber (AC). Various techniques have been proposed to facilitate graft unfolding in these cases [4,5,6,7,8,9,10,11,12].

Sutureless scleral fixation (SSF) has been developed to correct aphakia and address the complications associated with traditional sutured scleral-fixated IOL [13]. This approach includes methods such as the use of fibrin glue or tucking the haptics into scleral tunnels, as demonstrated in the Yamane technique, which involves the transconjunctival externalization of the haptics of a three-piece posterior chamber IOL. However, these techniques require greater surgical expertise and can present complications, including IOL tilt, haptic slippage, and IOL dislocation, partly due to the lack of IOLs specifically designed for this purpose [14,15]. To overcome these issues, the one-piece SSF IOL (FIL SSF, SOLEKO S.p.A., Pontecorvo, Italy), a hydrophilic acrylic IOL with T-shaped anchors designed for SSF, has been proposed recently [16,17]. 

Previous efforts have explored the combination of endothelial keratoplasty (EK) with SSF in one or two stages [18,19]. However, these attempts have been fraught with intraoperative and postoperative complications, including difficulties in graft unfolding, air dislocation, graft detachment, IOL opacification, and tilting [20,21]. Consequently, the reproducibility of these methods has been suboptimal.

Our study is the first to present a case series in which DMEK was performed in patients with SSF of one-piece IOL, whereas only a single case has been documented in the literature to date [5]. We introduce, for the first time in such complex cases, the air leak test as a crucial surgical step. This test enables the assessment of whether air, essential for securing the DMEK graft, is adequately retained within AC. In instances where the air was not contained, we reconstructed the iris-lens diaphragm through pupilloplasty.

## 2. Materials and Methods

This study was a retrospective, longitudinal, single-center, non-comparative case series that adhered to the principles outlined in the declaration of Helsinki and was approved by the Santa Croce e Carle Hospital Trial Clinical Center (n°38041). Clinical data were collected from 8 eyes of 8 patients who underwent DMEK surgery, having previously received SSF of one-piece IOL. For some patients, the decision to perform DMEK, either due to endothelial decompensation or failure of a previous DSAEK, was made after they had already undergone SSF of one-piece IOL implantation. For others who required both a secondary IOL implantation and an endothelial transplant, the two procedures were staged, with at least a 2-month interval between them.

**Figure 1:**
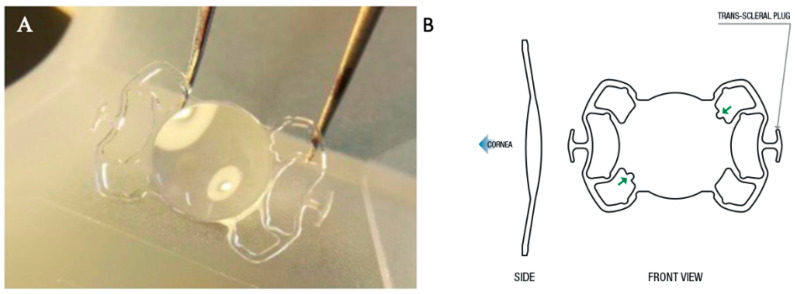
Photograph (**A**) and diagram (**B**) showing the structure of the new Carlevale sutureless scleral fixation intraocular lens (SSF-IOL). The arrow points to the two notches on the optic plate (upper right and lower left).

This foldable acrylic lens has two T-shaped self-blocking plugs on each haptic (angulation 10°), an optic diameter of 6.5 mm, and a total length of 13.2 mm, fitting through a 2.2 mm tunnel (Figure 1). Written informed consent was obtained from all patients.

The following data were collected: age, gender, indication for surgery, previous surgeries, preexisting ocular pathologies, time between SSF of one-piece IOL and DMEK surgery, and history of ocular trauma. All patients underwent a complete preoperative and postoperative ophthalmic examination at 3, 6, and 12 months. The follow-up period is defined as the time from DMEK surgery to the last visit, with a minimum duration of 12 months. Preoperative measurements of axial length (AXL) and AC depth (ACD) were performed using a swept source optical coherence tomography biometer (Argos^®^, Alcon Laboratories, Inc., Fort Worth, TX, USA) prior to DMEK surgery. At each visit, the central corneal thickness (CCT) was measured using anterior segment optical coherence tomography (AS-OCT, Visante, Carl Zeiss Meditec, Jena, Germany); the endothelial cell density (ECD), expressed as an average of measurements in several central/paracentral areas, was measured using an automated specular microscope (EM-3000, software version 1.2.2, Tomey Corporation, Osaka, Japan); the corrected distance visual acuity (CDVA) was assessed using the Revised 2000 Series ETDRS charts (Precision Vision, La Salle, IL, USA) and expressed in logMAR; subjective refraction was measured at 4 m and adjusted to infinity by subtracting 0.25 D [22]; and intraocular pressure (IOP) was measured by Goldmann applanation tonometry (Haag-Streit, Konig Switzerland) and with a Rebound Tonometer (ICare, Helsinki, Finland). Ultrasonography was performed on eyes with media opacity to exclude retinal pathology (HiScan Optikon 2000, BScan software version 3.1.10.11 Rome, Italy). The macula was assessed preoperatively with Spectralis OCT (Heidelberg Engineering, Heidelberg, Germany) when possible. Preoperative ECD data of the endothelial grafts were provided by the eye bank of the Piedmont region (Turin, Italy) and showed a preoperative value of approximately 2800 cells per square millimeter for all corneas. Yag laser iridotomy was performed at the 6 o’clock position if it had not been previously performed. The duration of the unfolding procedure was measured from the end of graft insertion into the AC to the start of gas injection underneath the graft; this was defined as the unfolding time. Furthermore, for each patient, the time between the SSF of one-piece IOL surgery and the DMEK was calculated.

### Surgery

All DMEK surgeries were performed on patients who had previously undergone SSF of one-piece IOL following complete vitrectomy with Balanced Salt Solution (BSS) tamponade as we previously described. 

The procedures were conducted using peribulbar anesthesia and intravenous sedation by the same experienced surgeon (A.S.V.). An 8 mm DMEK donor tissue was prepared using a double-punch technique. It was marked either with an inside triangle (cases 1 and 2) or with dry-ink gentian violet dye applied as an asymmetric ‘F’ mark on the stromal side of the DMEK tissue (cases 3–8). The graft was then stained with 0.06% trypan blue for 4 min before being loaded into a modified Jones tube with the endothelium facing outward. Cohesive viscoelastic was avoided to prevent incomplete removal in post-vitrectomized eyes or those with an altered iris-lens diaphragm. An AC maintainer regulated IOP and prevented hypotony during surgery [23,24,25].

After the removal of the recipient Descemet endothelium complex or DSAEK graft, miosis was induced with acetylcholine. Following this, the AC maintainer was removed, and an air leak test was performed by introducing air through a paracentesis to fill the entire AC. In cases where an air bubble was not confined but migrated posteriorly to the iris, pushing toward the cornea and effectively collapsing the chamber, the test was considered positive.

In such cases, after removing the air that had migrated behind the iris, we performed a pupilloplasty. This was carried out either to reduce the size of large iridotomies or to decrease the pupillary diameter, reconstructing a plane capable of containing the air bubble and preventing its posterior migration. This step was particularly important in irises affected by colobomas or large iridotomies, which often required single or multiple sutures. 

The stability of the iris plane was confirmed by reintroducing air into the chamber, ensuring that the bubble remained confined within it. For the pupilloplasty, a 10/0 Prolene™ suture (ref. 1713, Ethicon, New Brunswick, New Jersey, USA) was used. The technique involved the following steps: a straight needle was introduced through a paracentesis and passed through the free edge of the iris from top to bottom, and then from bottom to top on the opposite side. The needle was retrieved through the opposite paracentesis using a bent 25-gauge needle. An iris hook was used to pull the suture out of the original paracentesis. The suture was tied using either a Siepser knot or a single-pass 4-throw technique and finally trimmed using 23-gauge vitreous scissors. (Details on the air leak test and pupilloplasty can be found in Supplementary Video S1) [26,27].

**Figure 2:**
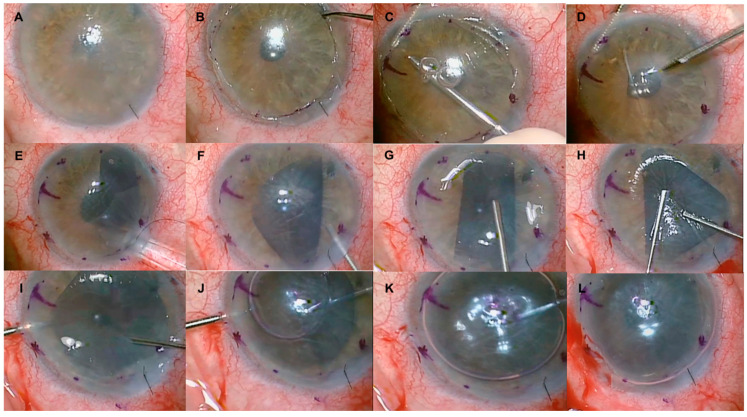
Clinical images showing the steps in centration and unfolding of a Descemet membrane endothelial keratoplasty graft within the anterior chamber in an eye with a sutureless scleral fixation intraocular lens placed in the posterior chamber. (**A**) Bullous keratopathy. (**B**) After removing the edematous corneal epithelium to improve visualization. (**C**,**D**) Scored and removed Descemet membrane and endothelium with a reverse sinskey hook following an 8.00 mm surface marking on the cornea centered at the pupil. (**E**) Corneal graft injected into the anterior chamber. (**F**–**H**) Centration and unfolding achieved by tapping on the top of the cornea with a cannula. (**I**–**K**) Serrated forceps securing the graft in place and preventing globe collapse during tapping. (**L**) A 20% sulfur hexafluoride gas bubble injected under the graft.

A 10/0 nylon suture was placed on the main incision, and after the AC maintainer was removed, the graft was injected into the AC, while keeping pressure low by releasing BSS through a paracentesis. Once opened even if only one side, the graft was gently grasped with a 23-gauge serrated forceps (Grieshaber; Alcon Laboratories, Fort Worth, Texas, USA.) for centration, and unfolding was achieved by tapping on the top of the cornea with cannula. After achieving proper positioning, a bubble of 20% sulfur hexafluoride (SF6) was injected very slowly while keeping the graft in place with the same forceps. The large AC means that, if the graft is not held in position, it may dislocate (details on graft positioning and gas injection can be found in Supplementary Video S2). All incisions were sutured with 10/0 nylon, and a bandage contact lens was placed if required. In all cases, subconjunctival dexamethasone (0.1%) and gentamicin (14 mg/mL) were injected. Figure 2 delineates the surgical procedures as outlined in the description. (Details of DMEK surgery can be found in Supplementary Video S3).

Postoperatively, patients were asked to remain in supine position for at least 3 h, and then the IOP was checked. In our standard DMEK protocol, we typically reduce the air in the AC, even when the IOP is within the normal range, allowing it to pass above a patent iridotomy or the lower edge of the dilated pupil. However, after performing this maneuver in the first patient (Case 1), the following day, we observed a partial graft detachment necessitating a rebubbling procedure. Consequently, in subsequent DMEK procedures for these complex cases, we opted not to decompress the AC, managing any mild ocular hypertension with medical treatment and supine position. As a result, we did not experience further graft detachments or the need for rebubbling. All patients received a standard postoperative regimen of moxifloxacin ophthalmic solution 0.5% t.i.d. for 1 week and dexamethasone sodium phosphate 0.1% every 3 h for 1 month, four times a day for 2 months, which was tapered down by one drop every 3 months to one drop daily for 1 year, and thereafter continued indefinitely. The suture was not removed until at least one month after surgery.

Statistical analyses were performed using MedCalc^®^ Statistical Software version 22.016. The distributions normality was assessed using the Shapiro–Wilk test. Data are shown as median (range). Wilcoxon tests were used to compare data. Statistical significance was set at *p* < 0.5.

## 3. Results

**Figure 3:**
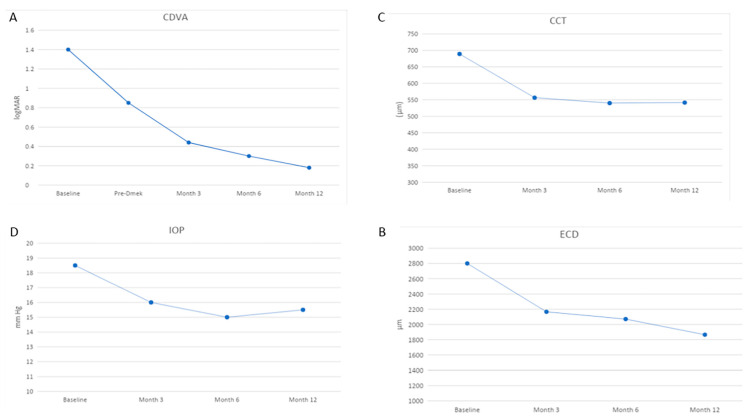
Four graphs showing changes during 12-month follow-up compared with baseline visit. (**A**) Median corrected distance visual acuity (CDVA) improvement. (**B**) Median corneal endothelial cell density (ECD) reduction. (**C**) Median central corneal thickness (CCT) trend. (**D**) Median intraocular pressure (IOP) trend.

**Figure 4:**
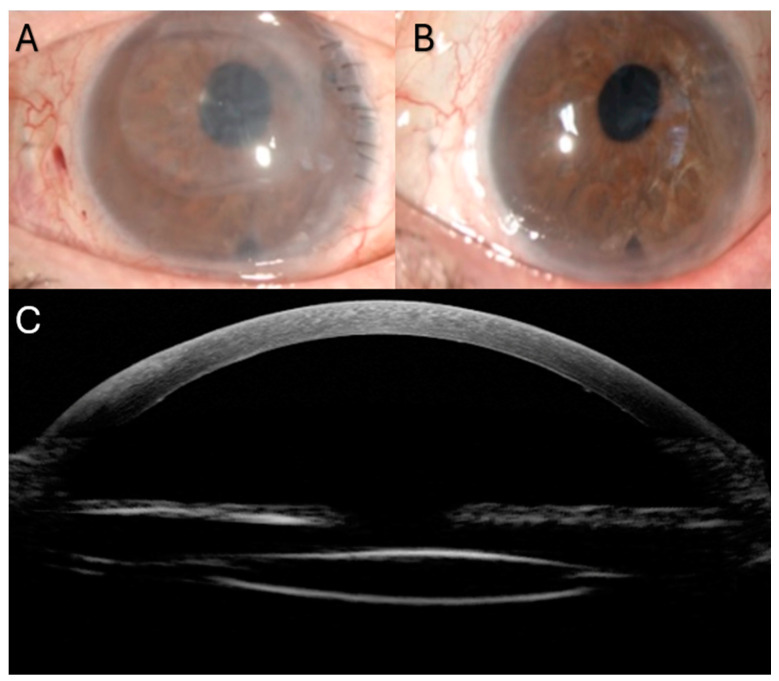
Case#2 of Descemet membrane endothelial keratoplasty (DMEK). (**A**) Slit-lamp color photograph at baseline and (**B**) at 3 months after DMEK surgery. (**C**) Reconstruction combining ultrasound biomicroscopy and anterior segment optical coherence tomography revealing both lens position and the attached DMEK graft 3 months after surgery.

Data from eight eyes of eight patients (six men and two women) were collected; the median age was 76.5 years (range: 53 to 85). Table 1 provides a summary of patient characteristics and morphological and functional outcomes. We performed pupilloplasty in four patients (50%). These cases included trauma (2), post-acute glaucoma (1), and post-anterior chamber IOL (1). The gas was stable within the AC at the end of surgery and did not migrate into the vitreous cavity in the postoperative period. Rebubbling for partial detachment was required in one patient (case 1) at day one postoperatively. Up to 12 months after the procedure, no complications, such as endophthalmitis, subluxation, IOL tilting, or opacifications, were observed. The median ACD was 3.5 mm (range: 3.04 to 5.06), median AXL was 24.2 (range: 23.7 to 25), and median IOL power was 23 (range: 19 to 25). The median CDVA improved significantly at each time point compared to 0.85 logMAR (range: 0.60 to 1.00) at baseline visit. It was 0.44 logMAR (range: 0.10 to 0.70, *p* = 0.012), 0.30 logMAR (range: 0.10 to 0.70, *p* = 0.011), and 0.18 logMAR (range: 0.10 to 0.70, *p* = 0.012) at 3, 6, and 12 months, respectively (Figure 3A). The median percentage reduction in ECD after 12 months was 33.4% (range: 30 to 40%). The median baseline CCT improved significantly over time, decreasing from 689 μm (range: 651 to 701) to 556.5 μm (range: 530 to 594, *p* = 0.008) at 3 months, 540 μm (range: 525 to 590, *p* = 0.012) at 6 months, and 541.5 μm (range: 525 to 591, *p* = 0.008) at 12 months (Figure 3C). The median follow-up time was 15 months (range: 12 to 25). Figure 4 shows ocular conditions before and after DMEK procedure.

## 4. Discussion

Descemet membrane endothelial keratoplasty is a well-established method of corneal transplantation for the treatment of corneal endothelial disease (CED). It is particularly suitable for patients with relatively clear stroma and easily visible iris details and offers several advantages, including minimal invasiveness with a “closed system” approach, which minimizes the risk of intraoperative vision-threatening complications, and it typically results in excellent postoperative visual outcomes [28]. Performing DMEK surgery on vitrectomized eyes, especially those with scleral lens fixation, presents unique challenges. These eyes are prone to a higher incidence of both intraoperative and postoperative complications [3]. In these eyes, lack of posterior support makes them susceptible to recurrent globe collapse during intraoperative manipulation. Additionally, they may have difficulty in shallowing the AC, which is a crucial step in DMEK surgery [29,30]. Finally, another important step is filling the AC with gas at the end of the procedure, so DMEK may be relatively contraindicated in eyes with unpressurized AC [31]. 

To perform DMEK in patients with vitrectomized eyes, on one hand, we adjusted the technique, and on the other, we sought to establish a stable iris-lens diaphragm. In post-vitrectomy eyes, tapping to unfold the graft can result in frequent inversion of the corneal curvature with globe collapse [2,3]. To enhance the stability of the AC and prevent this complication, some authors recommend posterior segment air infusion [8]. We applied external pressure on the sclera to maintain the convexity of the host cornea and, when necessary, a second surgeon supplemented the AC with a BSS injection through a paracentesis site.

As we mentioned, graft unfolding in eyes where it is difficult to flatten the AC was also more challenging than in standard DMEK procedures. To overcome these difficulties, some authors have developed the double-bubble technique. This technique involves the placement of two bubbles, one above and one beneath the graft, to facilitate the unfolding and fixation of the graft with ease and safety, demonstrating favorable clinical outcomes [6]. We are aware that prolonged manipulation to unfold the DMEK graft can lead to increased endothelial damage [2]. In our case series, because of the maneuver utilized for graft deployment, the median graft unfolding time was 6 min (range: 5 to 9). This duration is comparable to, or even shorter than, the time reported with the standard DMEK procedure [32]. Initially, tapping on the top of the cornea caused the graft to partially unroll on one side, facilitating the use of serrated forceps to secure it in place and prevent globe collapse during tapping. Subsequently, as the maneuver allowed the graft to be completely unrolled and positioned correctly with the endothelium facing downward, we continued to hold it while injecting a 20% SF6 gas bubble beneath the DMEK donor tissue. To ensure stability, it is essential to use serrated forceps or gradually release pressure from the top while introducing the gas. Failure to do so could result in the graft shifting and losing its correct position, particularly in eyes with a very deep AC, as indicated by our results, where the median ACD was 3.5 mm (range: 3.04 to 5.06), especially following vitrectomy and SSF IOL. As demonstrated by some authors, endothelial cell loss is considered negligible, even when multiple touches are required to position the graft during the procedure. This is because the tissue is grasped using only the distal ends of the forceps, thereby minimizing possible endothelial damage to an area of contact of almost 0.02 mm^2^ [33]. No evident tears were observed in the DMEK donor graft in any patient.

We established a stable iris-lens diaphragm using the one-piece SSF IOL, which provides four points of sulcus contact. Additionally, its large optic diameter of 6.5 mm ensures remarkable stability with minimal tilting [34]. Another crucial aspect was the reconstruction of the iris, because the presence of conditions like atrophic iris or iris defects, which are commonly encountered in such complex cases, compromised the effective separation of the anterior and posterior chambers, increasing the risk of complications such as graft migration, graft detachment, iris synechiae, IOL tilting, and opacification [3,31,35,36]. To address these challenges, we found the air leak test and the reconstruction of the iris-lens diaphragm to be very useful [16,37]. So, we performed pupilloplasty in four patients (50%). These cases included trauma (2), post-acute glaucoma (1), and post-AC IOL (1). All cases involved large iridectomies and atonic irises with compromised sphincter function.

In all cases, a complete vitrectomy was performed, and any rhegmatogenous retinal lesions present were treated with laser photocoagulation. In cases where pupilloplasty was performed, the size of the pupil did not interfere with the examination of the retinal periphery during follow-up visits. The large optic size of the IOL, when combined with the iris plane, establishes sufficient compartmentalization. This configuration forms a stable iris-lens diaphragm that consistently separates the anterior and posterior chambers. Consequently, it enables the eye to be pressurized effectively [38,39]. These measures, coupled with strict patient positioning in a supine posture postoperatively, ensured that the gas remained stable within the AC and did not migrate into the vitreous cavity in the postoperative period. The migration of gas into the posterior chamber lead to the formation of a 360° iridocorneal synechia, necessitating urgent intervention to dissolve the synechia and remove the gas from the vitreous chamber. The supine position is also crucial for postoperative management. In our standard DMEK protocol, after 3 h, we typically reduce the air in the AC to allow it to pass above a patent iridotomy or the dilated pupil, even when IOP is normal. However, after a partial graft detachment in the first case (case 1), which required rebubbling, we adjusted our approach for subsequent complex cases. We chose not to decompress the AC, instead managing mild ocular hypertension with medical treatment and continued supine positioning. In these eyes, the absence of vitreous and maintained positioning reduced the risk of pupillary block. This adjustment prevented further graft detachments or the need for rebubbling. In addition, we believe that a stable iris diaphragm minimizes the risk of IOL tilting and opacification. It is well known that air tamponade exerted onto the surface of the IOL poses a threat to its stability and clarity, potentially leading to subluxation or dislocation and IOL opacification, which can significantly impact visual acuity and quality, potentially necessitating further surgical intervention. This phenomenon is more common with certain types of IOL materials, such as hydrophilic acrylic lenses (such as FIL-SSF IOL). We did not observe any calcification of the IOL at almost 12 months postoperatively, despite the acrylic hydrophilic nature of the IOL. However, it is important to note that this complication cannot be completely ruled out [18]. 

In our opinion, DMEK may potentially provide better adaptation to irregular corneas [40] and offer the best chance for corneal clarity and visual recovery. Recently, a case report was published detailing DMEK combined with one-piece SSF IOL, demonstrating the IOL’s stability during indentation and tamponade, even under increased pressure in the AC due to the C-Press technique [5]. Both combined and sequential surgeries for patients requiring scleral fixation IOL and lamellar EK present postoperative complications [41,42]. 

While a combined procedure, such as the simultaneous performance of DMEK and SSF, offers the advantage that the patient follows a single fixed protocol of a postoperative drug regimen and undergoes fewer follow-ups compared to a staged method, it has been associated with a notable incidence of postoperative complications, including a high rate of rebubbling and significant endothelial cell loss, as corroborated by previous studies [37,38,39]. 

However, our observations align with those reported by Sinha et al. [43] highlighting the superior outcomes of staged surgery. Moreover, we believe that staged procedures offer several distinct advantages over simultaneous approaches. One notable benefit is the ability to execute the surgery through a smaller main incision, which makes the AC more stable for the DMEK surgery. Staged surgeries reduce the risk of intraoperative complications such as low intraocular pressure (hypotonia), residual vitreous in the AC, bleeding from the sclerotomy site, or iris damage, which may lead to fibrin formation. A two-step procedure provides a controlled and gradual approach, better inflammation management in the AC, surgical outcome optimization, and complication minimization [44,45]. 

Combined EK procedures are also associated with endothelial cell loss ranging from 3.3% to 27.7% depending on the intraoperative and postoperative course [37,40]. Recently, the outcomes of combining DSAEK with secondary IOL implantation have been compared to those of a two-stage procedure [44,45]. Short-term results favored the sequential approach, showing better visual outcomes, but no significant difference was observed after 6 to 9 months. Both groups had similar rates of primary graft failure and graft detachment [44,45]. Sequential surgery may offer immediate benefits by minimizing intraocular inflammation and potential complications associated with combined surgery [44,45]. 

Additionally, surgical maneuvers and the air pressure exerted onto the surface of the IOL can threaten its stability, potentially leading to the subluxation or dislocation of the newly implanted lens. In our sample, the median CDVA improved significantly from 0.85 logMAR (range: 0.60 to 1.00) at the baseline visit to 0.18 logMAR (range: 0.10 to 0.70, *p* = 0.012) at 12 months, indicating a substantial enhancement over the study period. Our visual outcomes are comparable with those of previous studies, as shown by the refractive outcomes [46] with a hyperopic shift from the median baseline refraction value of −1.00 diopter (range: −2.00 to 0.00) to −0.50 diopter (range: −0.75 to −1.00) pre- and postoperatively at 12 months, respectively. These results not only highlight the crucial stability of the lens for maintaining optimal visual acuity and quality over time, ensuring long-term satisfaction and functional vision for the patient but also strengthen the reliability and reproducibility of our findings. Additionally, our results show that the median percentage reduction in ECD after 12 months was 33.4% (range: 30 to 40%), which aligns with outcomes reported in other studies on DMEK combined with secondary IOL implantation. Similarly, the median baseline CCT of 689 μm (range: 651 to 701) decreased progressively to 556.5 μm (range: 530 to 594, *p* = 0.008) at 3 months, 540 μm (range: 525 to 590, *p* = 0.012) at 6 months, and 541.5 μm (range: 525 to 591, *p* = 0.008) at 12 months. This significant and consistent reduction in CCT, along with the marked improvement in corneal edema from baseline throughout the 12-month follow-up period, underscores the effectiveness and durability of the treatment approach, highlighting its potential for long-term success. The median postoperative IOP value at 12 months was 15.5 mm Hg (range: 12 to 18) and significantly changed from preoperative IOP values of 18.5 mm Hg (range: 15 to 21). In our series, no one patient had hypotony even in the early postoperative period. The reduced risk of wound leakage was attributed to the meticulous suturing of all access points to the AC during DMEK, as well as the decision to perform the procedure in a staged manner rather than combining it with other procedures.

The limitations related to the sample size are acknowledged, so larger long-term comparative studies are needed in the future to quantify the results and the complication rates of DMEK in eyes with SSF IOL even if anatomical and visual outcomes in this case series with a 12-month follow-up are very promising. Further studies may demonstrate that the air leak test and the reconstruction of the iris-lens diaphragm through pupilloplasty can be highly useful in patients who have undergone vitrectomy with secondary IOL implantation in the absence of the capsular bag and are candidates for DMEK. We used this DMEK technique exclusively with the SSF one-piece IOL, our preferred method for secondary IOL implantation, but while it still needs to be tested, with appropriate precautions, it could also be applied to other types of secondary IOL implantation and, in general, to vitrectomized eyes.

## 5. Conclusions

The effective compartmentalization of the anterior and posterior chambers in vitrectomized eyes with SSF one-piece IOL and pupilloplasty could facilitate the surgical steps of DMEK in these complex eyes. Additionally, the air leak test may be useful in patients undergoing DMEK with a history of vitrectomy and secondary IOL implantation, as it could indicate the need for iris-lens diaphragm reconstruction.

## Figures and Tables

**Table 1:** Patients details and surgical outcomes.

Median (Range) (Wilcoxon Test *p*-Value from Baseline)	CASE#1	CASE#2	CASE#3	CASE#4	CASE#5	CASE#6	CASE#7	CASE#8
Age (years) 76.5 (53-85)	79	68	80	62	78	85	53	75
Sex	M	M	M	M	F	F	M	M
Pathology	UT-DSAEK graft failure in previous acute glaucoma and complicated phacoemulsification	UT-DSAEK graft failure in previous AC IOL	dislocated IOL	aphakia post trauma	aphakia after complicated phacoemulsification	PEX dislocated IOL	UT-DSAEK graft failure in previous aphakia post trauma	aphakia after complicated phacoemulsification
Interval between suture less intrascleral one-piece IOL fixation and DMEK (months) 8 (2–33)	33	2	18	4	18	3	12	2
AC depth (µm) 3.5 (3.04–5.06) AXL 24.2 (23.7–25) IOL Power 23 (19–25) CDVA (log MAR) baseline after SSF IOL 0.85 (0.60 to 1.00)	3.1 24.2 23 1	3.04 23.83 18.5 0.6	3.2 23.9 23.5 0.7	4.15 25 24 1	3.2 23.94 25 0.6	5.06 23.74 19 0.7	3.7 24.2 21 1	3.5 23.7 23 1
3 months 0.44 (0.10 to 0.70)	0.4	0.1	0.18	0.7	0.3	0.48	0.6	0.48
6 months 0.30 (0.10 to 0.70)	0.4	0.1	0.18	0.7	0.3	0.18	0.3	0.3
12 months 0.18 (0.10 to 0.70)	0.4	0.1	0.18	0.7	0.18	0.1	0.3	0.18
Refraction (spherical equivalent) baseline after SSF IOL: −1.00 (−2.00 to 0.00)	−0.5	−2	0	−1	−1	−1	−0.5	−1.5
12 months: −0.50 (−0.75 to −1.00)	−0.5	−2	1	0	−0.75	−0.5	−0.5	−0.5
ECD (cells/mm²) baseline after SSF IOL	2800	2800	2800	2800	2800	2800	2800	2800
3 months 2165 (2000–2250)	2000	2100	2240	2250	2150	2150	2200	2180
6 months 2070 (1890–2180)	1900	1890	2180	2100	1950	2080	2120	2060
12 months 1865 (1680–1960)	1680	1780	1960	1850	1836	1920	1940	1880
CCT (µm) baseline after SSF IOL 689 (651–701)	688	668	651	701	694	690	675	694
3 months 556.5 (530–594)	571	594	530	560	568	538	553	541
6 months 540 (525–590)	555	590	526	545	540	540	525	540
12 months 541.5 (525–591)	560	591	526	545	542	541	525	540
IOP (mm Hg) baseline after SSF IOL 18.5 (15–21)	16	20	21	20	19	15	15	18
3 months 16 (14–18)	15	18	18	17	16	15	14	16
6 months 15 (10–18)	12	17	18	15	15	15	12	10
12 months 15.5 (12–18)	12	17	18	17	16	15	12	12
Pupilloplasty	yes	yes	no	yes	no	no	yes	no
Unfolding time (minutes) 6 (5–9)	5	9	7	6	8	5	6	6
Rebubbling Follow up (months). 15 (12–20)	Yes 12	No 14	No 12	No 18	No 12	No 17	No 16	No 20

AC = Anterior Chamber; CCT = Central Corneal Thickness; CDVA = Corrected Distance Visual Acuity; DMEK = Descemet Membrane Endothelial Keratoplasty; ECD = Endothelial Corneal Decompensation; IOL = Intraocular Lens; IOP= Intraocular Pressure; PEX = Pseudoexfoliation; SD = Standard Deviation; UT-DSAEK = Ultrathin Descemet Stripping Automated Endothelial Keratoplasty.

## Data Availability

Data are avaible in DigiSTAT archive Santa Croce & Carle hospital

## Supplementary Materials

The following supporting information can be downloaded at: https://www.mdpi.com/article/10.3390/jcm13226654/s1, Video S1 dmek carlevale. Video S2: air leak test and pupilloplasty. Video S3: graft positioning and gas Injection.
